# A *P*-value model for theoretical power analysis and its applications in multiple testing procedures

**DOI:** 10.1186/s12874-016-0233-0

**Published:** 2016-10-10

**Authors:** Fengqing Zhang, Jiangtao Gou

**Affiliations:** 1 Department of Psychology, Drexel University, 3201 Chestnut Street, Philadelphia, 19104 USA; 2 Department of Mathematics and Statistics, Hunter College of CUNY, 695 Park Avenue, New York, 10065 USA

**Keywords:** Critical constants, Multiple testing procedures, Power analysis, *p*-value

## Abstract

**Background:**

Power analysis is a critical aspect of the design of experiments to detect an effect of a given size. When multiple hypotheses are tested simultaneously, multiplicity adjustments to *p*-values should be taken into account in power analysis. There are a limited number of studies on power analysis in multiple testing procedures. For some methods, the theoretical analysis is difficult and extensive numerical simulations are often needed, while other methods oversimplify the information under the alternative hypothesis. To this end, this paper aims to develop a new statistical model for power analysis in multiple testing procedures.

**Methods:**

We propose a step-function-based *p*-value model under the alternative hypothesis, which is simple enough to perform power analysis without simulations, but not too simple to lose the information from the alternative hypothesis. The first step is to transform distributions of different test statistics (e.g., *t*, chi-square or *F*) to distributions of corresponding *p*-values. We then use a step function to approximate each of the *p*-value’s distributions by matching the mean and variance. Lastly, the step-function-based *p*-value model can be used for theoretical power analysis.

**Results:**

The proposed model is applied to problems in multiple testing procedures. We first show how the most powerful critical constants can be chosen using the step-function-based *p*-value model. Our model is then applied to the field of multiple testing procedures to explain the assumption of monotonicity of the critical constants. Lastly, we apply our model to a behavioral weight loss and maintenance study to select the optimal critical constants.

**Conclusions:**

The proposed model is easy to implement and preserves the information from the alternative hypothesis.

## Background

Power analysis is a key technique in the experimental design to reveal an effect of a given size. Traditional power calculation usually assumes a single hypothesis test, but it is quite common for researchers to test several hypotheses simultaneously. Clinical trials often require two or more hypotheses to be tested, and studies which involve comparing treatments using multiple outcome measures happen frequently in medical research [8]. The development of high-throughput biology leads to a dramatic increase in the number of hypothesis tests in genomics [20]. However, there are a limited number of studies on power analysis in multiple testing procedures. For scientific studies with multiple hypotheses, in order to correctly control the false positives, multiplicity adjustments to *p*-values should be taken into account in power analysis. A consequence of multiplicity adjustments is the loss of power [21] and the change in sample size requirements [16].

The *p*-value is a tail probability given the null hypothesis is true. Under the null hypothesis, the *p*-value is a uniformly distributed random variable between 0 and 1. If the null hypothesis is false, the *p*-value’s distribution depends on the alternative hypothesis, which usually satisfies the inequality

1 $$ \Pr(P \leq p) \geq p,  $$

i.e., the random variable *P* is less than a standard uniform random variable in the stochastic order, where the random variable *P* is the probability of rejecting the null hypothesis when the alternative hypothesis is true.

In order to perform a *p*-value based power analysis, certain distribution models are needed to describe the behavior of *p*-values under the alternative hypothesis. In general there are two approaches. The *p*-value models based on the original test statistics [17] or based on copulas [27] are usually with complex expressions, and further calculations or evaluations require integrations. So the theoretical analysis is difficult and numerical simulations are often needed. The *p*-value models based on Dirac function [10, 24] are over-simplified, which limits their application areas.

In this paper, we propose the step-function-based *p*-value models under the alternative hypothesis, which are simple enough to perform theoretical power analysis, but not too simple to lose the information from the alternative hypothesis. Two applications in multiple testing procedures are shown and one application in weight-loss treatment is given.

## Methods

A widely used *p*-value model assumes that the statistic under the null hypothesis follows *N*(0,1^2^), and the statistics under the alternative hypothesis follows *N*(*δ*,1^2^). The *p*-values are calculated based on one-sided test [17].

The density function of the normal-distribution-based *p*-value model is

2 $$ h(p) = \frac{\phi\left(\Phi^{-1}(p) + \delta\right)}{\phi\left(\Phi^{-1}(p)\right)},  $$

with mean and variance

$$\begin{array}{*{20}l} \mathrm{E}[P] & =\int_{-\infty}^{+\infty} \Phi(x)\phi\left(x + \delta\right) dx, \\ \text{var}[P] &= \int_{-\infty}^{+\infty} \left[\Phi(x)\right]^{2}\phi\left(x + \delta\right) dx \\ & \quad - \left(\int_{-\infty}^{+\infty} \Phi(x)\phi\left(x + \delta\right) dx\right)^{2}. \end{array} $$

Alternatively, we propose a step-function-based *p*-value model under the alternative hypothesis, which has a density function

3 $$ h(p) = \left\{ \begin{aligned} f, &\quad 0\leq p \leq \frac{1-g}{f-g},\\ g, &\quad \frac{1-g}{f-g} < p \leq 1, \end{aligned}\right.  $$

with mean and variance

$$\begin{aligned} {}\mathrm{E}[P] = \frac{1 - 2g + fg}{2\left(f-g\right)}, \quad \text{var}[P] = \frac{1 + 4fg^{2} + 4f^{2}g - 3f^{2}g^{2}}{12\left(f-g\right)^{2}}, \end{aligned} $$

where 0≤*g*≤1≤*f*. The parameter (*f*,*g*) indicates the deviation of the random variable *P* under the step-function-based *p*-value model from a standard uniform random variable.

For comparison between the normal-distribution-based *p*-value model and the step-function-based *p*-value model, the parameters (*f*,*g*) of the step-function-based *p*-value model were chosen to match the means and the variances for the normal-distribution-based *p*-value model with parameter *δ*, as shown in Table 1. For the simplified step-function-based *p*-value model with parameter *f*, means are matched with the normal model.

**Table 1:** Normal-distribution-based *p*-value model and step-function-based *p*-value model

Normal			Step-function			Simplified step-function	
*δ*	Mean	SD	*f*	*g*	(1−*g*)/(*f*−*g*)	*f*	1/*f*
0.2	0.444	0.286	1.249	0.795	0.451	1.127	0.888
0.5	0.362	0.274	1.728	0.555	0.380	1.382	0.724
1	0.240	0.236	2.937	0.288	0.269	2.086	0.480
2	0.079	0.130	9.061	0.059	0.105	6.357	0.157
3	0.017	0.049	37.143	0.007	0.027	29.503	0.034

In addition, a simplified step-function-based *p*-value model with a single parameter *f*∈[1,+*∞*) is achieved when assuming *g*=0. The corresponding density function is

4 $$ h_{0}(p) = \left\{ \begin{aligned} f, &\quad 0\leq p \leq \frac{1}{f},\\ 0, &\quad \frac{1}{f} < p \leq 1. \end{aligned}\right.  $$

with mean and variance

$$ \mathrm{E}[P] = \frac{1}{2f}, \quad \text{var}[P] = \frac{1}{12f^{2}}. $$

**Fig. 1:**
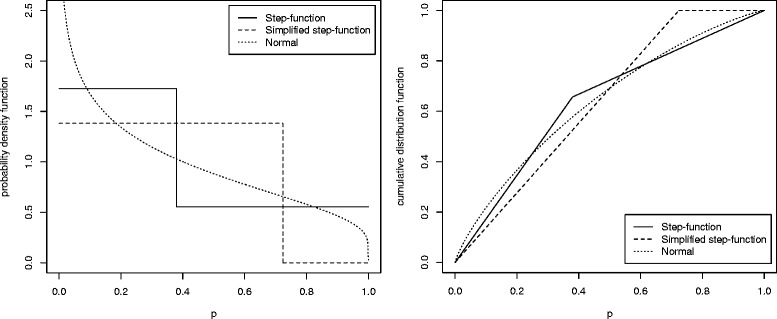
Normal-distribution-based and step-function-based *p*-value models (*δ*=0.5)

For the simplified step-function-based *p*-value model, a larger parameter *f* corresponds to a larger effect size. As a special case, when *f*=1, the distribution is uniform [0,1]. The probability density functions and the cumulative distribution functions of the normal-distribution-based and the step-function-based *p*-value models are compared in Fig. 1. The step-function-based *p*-value models serve an approximation to the *p*-value models based on the original test statistics.

Based on the univariate model, the corresponding multivariate *p*-value model has a density function

5 $$ h(p_{1}, \cdots, p_{n}) = \prod_{i=1}^{n} h_{i}\left(p_{i}\right),  $$

where different *h*_*i*_(·)’s may have different parameters *f*_*i*_’s and *g*_*i*_’s. In the following sections, the simplified step-function-based *p*-value model is applied to two problems in multiple testing procedures.

## Results

### Application: optimal choices of the critical constants

Assume *n* hypotheses $\left \{H_{i}\right \}_{i=1}^{n}$ with *p*-values $\left \{p_{i}\right \}_{i=1}^{n}$. Sort *p*-values as *p*_(1)_≤⋯≤*p*_(*n*)_, and *H*_(1)_,⋯,*H*_(*n*)_ are the corresponding null hypotheses. Consider testing the global null hypothesis $\cap _{i=1}^{n} H_{i}$ under the control of type I error [15]

$$ \Pr\left(\cup_{i=1}^{n} \left\{p_{(n-i+1)} \leq c_{i} \alpha \right\}\right) \leq \alpha. $$

The global test compares *p*_(*n*−*i*+1)_ with its corresponding critical constant *c*_*i*_ *α* for every *i*=1,⋯,*n*. If for some *i*’s, *p*_(*n*−*i*+1)_≤*c*_*i*_ *α*, then the global null hypothesis is rejected. Simes [25] proposed a test with *c*_*i*_=(*n*−*i*+1)/*n*, and other choices of *c*_*i*_’s were proposed by Rom [22], Cai and Sarkar [7], Gou and Tamhane [11].

Among different choices of critical constants *c*_*i*_’s, people usually run simulations [19] or rely on numerical calculations [14] to make power comparison in order to choose suitable sets of critical constants. Besides the existing computationally intensive methods, the step-function-based *p*-value model is an alternative choice to theoretically calculate the powers and make comparisons between different multiple testing procedures.

In this section we first show how the most powerful critical constants can be chosen using our proposed method for a global test with two hypotheses, and then we can apply the successive recursion process to calculate the power for a global test with *n* hypotheses. For a global test with two single hypotheses, the control of type I error under independence requires

$$ \Pr\left(\left\{p_{(2)} \leq c_{1} \alpha \right\} \cup \left\{p_{(1)} \leq c_{2} \alpha \right\}\right) = \alpha, $$

where *c*_1_≥*c*_2_. This equality is equivalent to

6 $$ \left(1 - 2c_{2}\right) + c_{1}\left(2c_{2} - c_{1}\right)\alpha = 0.  $$

From (6) we get $c_{2} = \frac {1 - {c_{1}^{2}}\alpha }{2\left (1 - c_{1} \alpha \right)}$, and the derivative $\frac {d c_{2}}{d c_{1}} = - \frac {\left (c_{1} - c_{2}\right)\alpha }{1 - c_{1}\alpha }$, which is less than zero. So *c*_2_ decreases when *c*_1_ increases.

By using the simplified step-function-based *p*-value model, without loss of generality, we assume 1≤*f*_1_≤*f*_2_, the probability of rejecting the global hypothesis is

$$ {}\begin{aligned} &\text{Rejection probability} \\ &= \left\{ \begin{array}{ll} c_{2}\left(f_{1} + f_{2}\right)\alpha + c_{1} \left(c_{1} - 2c_{2}\right) f_{1}f_{2}\alpha^{2}, & \quad \text{when}\,\, f_{1} \leq f_{2} \leq \frac{1}{c_{1}\alpha}, \\ \left(c_{1}f_{1} + c_{2}f_{2}\right)\alpha - c_{1}c_{2}f_{1}f_{2}\alpha^{2}, &\quad \text{when}\,\, f_{1} \leq \frac{1}{c_{1}\alpha} \leq f_{2} \leq \frac{1}{c_{2}\alpha},\\ 1, &\quad \text{when}\,\, f_{1} \geq \frac{1}{c_{1}\alpha} \text{or} f_{2} \geq \frac{1}{c_{2}\alpha}. \end{array}\right. \end{aligned}  $$

When the global hypothesis contains two single hypotheses (*n*=2), there are two configurations of true and non-true null hypotheses where the global null hypothesis is false: (1) one true null hypothesis (*n*_0_=1) and one false null hypothesis (*m*=1), and (2) two false null hypotheses (*m*=2).

First, assume that one null hypothesis is true and the other is false, say, *f*_1_=1 and *f*_2_=*f*, then the power is

$${\kern-16.5pt}\begin{aligned} \text{Power} = \left\{ \begin{array}{ll} c_{2} \left(1 + f\right) \alpha + fc_{1}\left(c_{1} - 2 c_{2}\right)\alpha^{2}&\quad \text{when}\,\, f \leq \frac{1}{c_{1}\alpha}, \\ \left(c_{1} + fc_{2}\right)\alpha - fc_{1} c_{2} \alpha^{2}, &\quad \text{when}\,\, \frac{1}{c_{1}\alpha} \leq f \leq \frac{1}{c_{2}\alpha}, \\ 1, &\quad \text{when}\,\, f \geq \frac{1}{c_{2}\alpha}. \end{array}\right. \end{aligned} $$

When $f \leq \frac {1}{c_{1}\alpha }$, from (6) it follows that *c*_1_(*c*_1_−2*c*_2_)*α*=1−2*c*_2_, therefore

$$c_{2} \left(1 + f\right) \alpha + fc_{1}\left(c_{1} - 2 c_{2}\right)\alpha^{2} = \left(f - \left(f - 1\right)c_{2}\right)\alpha, $$

hence power increases when *c*_2_ decreases (*c*_1_ increases).

When $\frac {1}{c_{1}\alpha } \leq f \leq \frac {1}{c_{2}\alpha }$, from (6) we get $c_{1} c_{2} \alpha = \frac {1}{2}\left (2c_{2} - 1 + {c_{1}^{2}}\alpha \right)$, consequently

$${}\begin{aligned} \left(c_{1} + fc_{2}\right)\alpha - fc_{1} c_{2} \alpha^{2} &= \frac{\alpha}{2}\left(f + 2 c_{1} - f\alpha {c_{1}^{2}}\right) \\ &= \frac{1}{2f}\left(1 + f^{2}\alpha - \left(f\alpha c_{1} - 1\right)^{2}\right). \end{aligned} $$

Note that *f* *α* *c*_1_≥1, so power increases when *c*_1_ decreases (*c*_2_ increases).

We calculate the maximal power for different *f*’s. When we assume the alternative hypothesis has a small effect size, where $f \leq 1/\sqrt {\alpha }$, the maximal power is achieved when *c*_2_=0 and $c_{1} = 1/\sqrt {\alpha }$. When we assume the alternative hypothesis has a moderate effect size, where $1/\sqrt {\alpha } < f \leq \left (1 + \sqrt {1 - \alpha }\right)/\alpha $, the maximal power is achieved when *c*_1_=1/(*f* *α*) and *c*_2_=(*f* ^2^ *α*−1)/(2*α* *f*(*f*−1)), so when *f* increases, we can follow the strategy to decrease *c*_1_ (increase *c*_2_) to achieve the maximal power. When we assume the alternative hypothesis has a large effect size, where $f > \left (1 + \sqrt {1 - \alpha }\right)/\alpha $, the maximal power is achieved when we choose a test with *c*_2_≥1/(*f* *α*), so when *f* is large enough, different tests have similar power.

7 $$\begin{array}{*{20}l} \max\text{Power} = \left\{ \begin{array}{ll} f\alpha, &\quad \text{when}\,\, f \leq \frac{1}{\sqrt{\alpha}}, \\ \frac{1 + f^{2}\alpha}{2f}, &\quad \text{when}\,\, \frac{1}{\sqrt{\alpha}} < f \leq \frac{1 + \sqrt{1 - \alpha}}{\alpha}, \\ 1, &\quad \text{when}\,\, f > \frac{1 + \sqrt{1 - \alpha}}{\alpha}. \end{array}\right. \end{array} $$

Second, assume that both null hypothesis are false with the same effect size, say, *f*_1_=*f*_2_=*f*, then the power is

$${}\begin{aligned} \text{Power}= \left\{ \begin{array}{ll} 2fc_{2}\alpha + f^{2} c_{1} \left(c_{1} - 2c_{2}\right) \alpha^{2}, &\quad \text{when}\,\, f \leq \frac{1}{c_{1}\alpha}, \\ 1, &\quad \text{when}\,\, f \geq \frac{1}{c_{1}\alpha}. \end{array}\right. \end{aligned} $$

When $f \leq \frac {1}{c_{1}\alpha }$, from (6) we get *c*_1_(*c*_1_−2*c*_2_)*α*=1−2*c*_2_, then

$$2fc_{2}\alpha + f^{2} c_{1} \left(c_{1} - 2c_{2}\right) \alpha^{2} = f \alpha \left(f - 2\left(f - 1\right)c_{2}\right), $$

power increases when *c*_2_ decreases (*c*_1_ increases).

We calculate the maximal power for different *f* values. When we assume both hypotheses are false and with a small effect size, where $f \leq 1/\sqrt {\alpha }$, the maximal power is achieved when *c*_2_=0 and $c_{1} = 1/\sqrt {\alpha }$. When we assume both false hypotheses have a big effect size, where $f \geq 1/\sqrt {\alpha }$, the maximal power is achieved when the test satisfies *c*_1_≥1/(*f* *α*). By taking both $f\leq 1/\sqrt {\alpha }$ and $f \geq 1/\sqrt {\alpha }$ into account, it follows that $c_{1} = 1/\sqrt {\alpha }$ and *c*_2_=0 is the uniformly best choice when both null hypotheses are false.

8 $$\begin{array}{*{20}l} \max\text{Power} = \left\{ \begin{array}{ll} f^{2}\alpha, &\quad \text{when}\,\, f \leq \frac{1}{\sqrt{\alpha}}, \\ 1, &\quad \text{when}\,\, f > \frac{1}{\sqrt{\alpha}}. \end{array}\right. \end{array} $$

In general, for a global test with *n* single hypotheses, where *m* of them are true significances (false null hypotheses), define the probabilities as

$${}\begin{aligned} B_{n,m,i} \\ &{}= \left\{ \begin{array}{ll} {\Pr}_{n,m}\left(p_{(n)} \leq c_{1}\alpha\right), &\,\,\, i=1,\\ {\Pr}_{n,m}\left(p_{(n)} > c_{1}\alpha, \cdots, p_{(n-i+2)} > c_{i-1}\alpha, p_{(n-i+1)} \leq c_{i}\alpha\right), &\,\,\, i=2,\cdots, n, \\ {\Pr}_{n,m}\left(p_{(n)} > c_{1}\alpha, \cdots, p_{(1)} > c_{n}\alpha\right), &\,\,\, i=n+1, \end{array}\right. \end{aligned} $$

where Pr_*n*,*m*_ indicates the probability for *n* hypotheses, where *m* is the number of the true significances, and *n*_0_=*n*−*m* is the number of the true nulls.

Since

$${}\begin{aligned} B_{n,m,n} &= {\Pr}_{n,m} \left(p_{(n)} > c_{1}\alpha, \cdots, p_{(2)} > c_{n-1}\alpha, p_{(1)} \leq c_{n}\alpha\right) \\ &= \left(n-m\right){\Pr}_{n-1,m} \left(p_{(n-1)} > c_{1}\alpha, \cdots, p_{(1)}\right.\\ &\quad \left. > c_{n-1}\alpha\right) {\Pr}_{1,0}\left(p \leq c_{n}\alpha\right) \\ &\quad + m {\Pr}_{n-1,m-1} \left(p_{(n-1)} > c_{1}\alpha, \cdots, p_{(1)} \right.\\ &\quad \left.> c_{n-1}\alpha\right) {\Pr}_{1,1}\left(p \leq c_{n}\alpha\right), \end{aligned} $$

we have the recurrence relation for *i*=*n*

9 $$ B_{n,m,n} = (n-m) c_{n}\alpha B_{n-1, m,n} + m \left(fc_{n}\alpha \wedge 1\right) B_{n-1, m -1,n}.  $$

Similarly, for general *i*, since

$$\begin{array}{*{20}l} B_{n,m,i} &= {\Pr}_{n,m}\left(p_{(n)} > c_{1}\alpha, \cdots, p_{(n-i+2)}\right.\\ & \quad \left. > c_{i-1}\alpha, p_{(n-i+1)} \leq c_{i}\alpha\right) \\ &= \tfrac{n-m}{n-i+1}{\Pr}_{n-1,m}\left(p_{(n-1)} > c_{1}\alpha, \cdots, p_{(n-i+1)}\right.\\ & \quad \left. > c_{i-1}\alpha, p_{(n-i)} \leq c_{i}\alpha\right) {\Pr}_{1,0}\left(p \leq c_{i}\alpha\right) \\ & \quad + \tfrac{m}{n-i+1} {\Pr}_{n-1,m-1}\left(p_{(n-1)} > c_{1}\alpha, \cdots\!, p_{(n-i+1)} \right.\\ & \quad \left. > c_{i-1}\alpha, p_{(n-i)} \leq c_{i}\alpha\right) {\Pr}_{1,1}\left(p \leq c_{i}\alpha\right), \end{array} $$

we have the general recurrence relation for *i*

10 $$ \begin{aligned} B_{n,m,i} &= \frac{n-m}{n-i+1} c_{i}\alpha B_{n-1, m,i}\\ & \quad + \frac{m}{n-i+1} \left(fc_{i}\alpha \wedge 1\right) B_{n-1, m-1, i}. \end{aligned}  $$

Finner and Roters [9], Cai and Sarkar [7], and Gou and Tamhane [11] defined a special case of the probability *B*_*n*,*m*,*i*_ for *m*=0 to calculate the type I error under the global null hypothesis. They proved a recurrence relationship among *B*_*n*,0,*i*_’s. We generalize this result to *B*_*n*,*m*,*i*_’s and have this recurrence relationship (10) under the simplified step-function-based *p*-value model for power analysis.

By starting from

$$\begin{array}{*{20}l} B_{1,0,1} &= c_{1}\alpha, & B_{1,0,2} &= 1 - c_{1} \alpha, \\ B_{1,1,1} &= fc_{1}\alpha \wedge 1, & B_{1,0,2} &= 1 - {fc}_{1} \alpha \wedge 1, \end{array} $$

and using the recurrence relation (10), the power is calculated by

11 $$ \text{Power}_{n,m} = \sum_{i=1}^{n} B_{n,m,i}  $$

Note that

$$ \sum_{i=1}^{n+1} B_{n,m,i} = 1, $$

and the control of type I error is satisfied if

$$ \sum_{i=1}^{n} B_{n,0,i} \leq \alpha. $$

When *f* is specified and the set of critical constants $\left \{c_{i}\right \}_{i=1}^{n}$ is given, the exact power can be calculated by using (11). Since only arithmetic calculations are needed, the power can be computed very fast.

For theoretical analysis, we consider the situation where *f* is not too small.

$$ \text{Power}_{n,m} = 1, \quad \text{when}\,\, f \geq \frac{1}{c_{n+1-m}\alpha}. $$

The largest possible *c*_*n*+1−*m*_ is achieved by using the set of critical constants which satisfies *c*_1_=*c*_2_=⋯=*c*_*n*+1−*m*_=*c*, and *c*_*n*−*m*+2_=⋯=*c*_*n*_=0. The control of type I error requires that

12 $$ \sum_{i=0}^{n-m} {n \choose i} \left(c\alpha\right)^{n-i} \left(1 - c\alpha\right)^{i} \leq \alpha,  $$

the largest possible *c*_*n*+1−*m*_ can be solved from (12). If we only take the leading term, we have an approximate solution

$$ c_{n+1-m} \approxeq \frac{1}{\sqrt[m]{{n\choose m} \alpha^{m-1}}} $$

So when

$$ f \gtrapprox \sqrt[m]{\tfrac{{n \choose m}}{\alpha}} $$

the maximal power is achieved when the test satisfies *c*_*n*+1−*m*_≥1/(*f* *α*).

Note that when *m* is relatively large (e.g., more than *n*/2), the bound $\sqrt [m]{{{n \choose m}}/{\alpha }}$ is small, and the global tests with large *c*_1_,⋯,*c*_*n*+1−*m*_ and small *c*_*n*+2−*m*_,⋯,*c*_*n*_ tend to have large power. Similar observations were reported by Gou and Tamhane [11] based on simulations.

Note that in this application power is simply the probability of rejecting $H_{0} = \cap _{i=1}^{n} H_{i}$ where at least one *H*_*i*_ is false. For testing multiple hypothesis, powers can be of different types: individual, average, disjunctive, and conjunctive, and the appropriate power concept is determined on a case-by-case basis [4]. These power definitions can also be used in the proposed method by using the step-function-based *p*-value model.

Power analysis can be complex when multiple hierarchical objectives are involved. Alosh and Huque [1] discussed the power for testing hierarchically ordered endpoints. The step-function-based *p*-value model can be applied to various clinical trials, e.g., group sequential designs [18], graphical procedures [5, 6].

#### Application: monotonicity of the critical constants

For multiple test procedures, critical constants are often required to satisfy [7, 12]

13 $$ c_{1} \geq c_{2} \geq \cdots \geq c_{n}  $$

This requirement is called the monotonicity assumption of critical constants.

Suppose that we have a set of critical constants $c_{1}^{*}, \cdots, c_{n}^{*}$, and $c_{k}^{*} < c_{k+1}^{*}$, so the monotonicity assumption is not satisfied. Note that

$${}\begin{aligned} \Pr\left(\!\!{\vphantom{\cup_{i=1, i \neq k}^{n}}}\cup_{i=1}^{n}\left\{p_{(n+1-i)} \leq c_{i}^{*}\alpha \right\}\!\right) &= \Pr\left(\!\cup_{i=1, i \neq k}^{n}\left\{p_{(n+1-i)} \right.\right.\\ & \quad \!\left.\left.\leq c_{i}^{*}\alpha \!\right\}\! \cup \!\left\{\! p_{(n+1-k)} \!\leq\! c_{k+1}^{*}\alpha\right\}\!\!{\vphantom{\cup_{i=1, i \neq k}^{n}}}\right) \end{aligned} $$

So if a test with critical constants $c_{1}^{*}, \cdots, c_{k-1}^{*}, c_{k}^{*}, c_{k+1}^{*}, \cdots, c_{n}^{*}$ controls type I error below *α*, then another test with critical constants $c_{1}^{*}, \cdots, c_{k-1}^{*}, c_{k+1}^{*}, c_{k+1}^{*}, \cdots, c_{n}^{*}$, which satisfies the monotonicity assumption, also controls type I error below *α*, and has the same power with the previous test which does not satisfy the monotonicity assumption. Hence, only the set of critical constants which satisfies the monotonicity assumption needs to be considered.

Many multiple tests have critical constants which satisfy a strict monotonicity assumption [11, 22, 25]

14 $$ c_{1} > c_{2} > \cdots > c_{n}.  $$

Some multiple tests satisfy the monotonicity assumption (13), but do not satisfy the strict monotonicity assumption (14) [3, 26]. In general, these tests are not as powerful as the tests which satisfy the strict monotonicity assumption (14) [11]. Our step-function-based *p*-value models can explain that this assumption is necessary because the corresponding tests are generally more powerful than other tests which do not satisfy this assumption.

For multiple tests with two single hypotheses, by using the simplified step-function-based *p*-value model, we have several observations: (1) when there is one true null hypothesis and one false null hypothesis, only if $f = \left (1 + \sqrt {1-\alpha }\right)/\alpha $, the test which does not satisfy (14) can be more powerful than or as powerful as all the tests which satisfy (14), (2) when there are two false null hypotheses, the test does not satisfy (14) is less powerful than some of the tests which satisfy (14) for all *f* values. In general, on the parameter space of the effect size (under simplified step-function-based *p*-value model, the effect size is a function of parameter *f*), the tests which do not satisfy (14) have more power than all other tests which satisfy (14) only at a zero measure subspace of the effect size. This fact explains that usually people prefer multiple tests which satisfy the strict monotonicity property (14), because these tests are generally more powerful than the tests which do not satisfy (14).

#### A worked example

Annesi et al. [2] evaluated behavioral weight-loss treatments. They recruited 110 women whose BMI’s are between 30 and 40 kg/m^2^, and randomly assigned the participants to a comparison treatment with a print manual and telephone follow-ups, or an experimental treatment of the coach approach exercise-support protocol. The self-efficacy for controlled eating (SE-eating) is one of the psychological predictors of behavioral changes. Annesi et al. [2] reported that during the weight-loss phase (month 0-6), the SE-eating increases in the experimental group were significantly greater than the increases in the comparison group with *t*=2.88, and there was no significant between-group difference during the weight-loss maintenance phase (month 6-24) with *t*=−0.48.

The increases of the self-efficacy for controlled eating were evaluated both during the weight-loss phase and during the weight-loss maintenance phase, so the multiplicity adjustment is advised to apply. To choose the optimal multiplicity correction based on the estimated effect size from the pilot study, we recommend our proposed step-function-based *p*-value model because it is easy to implement and preserves the information from the alternative hypothesis. If we take the weight-loss study by Annesi et al. [2] as a pilot study, we have the information that the standardized SE-eating increase during the weight-loss phase is normally distributed with mean *δ*=2.88 and variance 1, and the increase during the weight-loss maintenance phase is normally distributed with mean *δ*=0 and variance 1. To match the mean for the normal-distribution-based *p*-value model with parameter *δ*=2.88, the parameter *f* of the simplified step-function-based *p*-value model is 23.979. From (7) and by using the significance level *α*=0.05, the maximal power is (1+*f* ^2^ *α*)/(2*f*)=62 *%*, and the optimal choice of critical constants is (*c*_1_,*c*_2_)=(1/(*f* *α*),(*f* ^2^ *α*−1)/(2*α* *f*(*f*−1)))=(0.8341,0.5036). So the larger *p*-value is compared with 0.8341*α* and the smaller *p*-value is compared with 0.5036*α*, and if any *p*-value is less than the corresponding critical value, the global null hypothesis will be rejected.

The step-function-based *p*-value models for power analysis simplify the theoretical analysis that is difficult in many situations. At the same time, information of loss remains at an acceptable level. Finner and Gontscharuk [10] and Sarkar et al. [24] used a tool called the Dirac–uniform configuration for power analysis, where all *p*-values under the false null hypotheses follow a Dirac distribution with point mass at 0. When the Dirac-uniform configuration is applied to Annesi et al.’s [2] study, the information of *δ* is lost, and any choice of positive critical constants (*c*_1_,*c*_2_) will result a claim of significance. Under the *p*-value model based on Dirac function, all choices of critical constants have the same power, and the optimal choice is unable to be located.

The Dirac-uniform configuration is too brief to include necessary information to choose the optimal critical constants. Hung et al. [17] discussed a *p*-value model based on normal distribution. By using Annesi et al.’s [2] research as a pilot study, one test statistic is *N*(*δ*,1^2^), and the other test statistic is *N*(0,1^2^). The probability of rejecting the global null hypothesis is

$$\begin{array}{*{20}l} \text{Power} &= \left[ \int_{-\infty}^{\Phi^{-1}(c_{1}\alpha)} \int_{-\infty}^{\Phi^{-1}(c_{1}\alpha)} + \int_{-\infty}^{\Phi^{-1}(c_{2}\alpha)} \int_{\Phi^{-1}(c_{1}\alpha)}^{+\infty}\right.\\ & \quad \left. + \int_{\Phi^{-1}(c_{1}\alpha)}^{+\infty} \int_{-\infty}^{\Phi^{-1}(c_{2}\alpha)} \right] \\ &\qquad \phi(x_{1} + \delta) \phi(x_{2}) \;d x_{1} d x_{2}\\ & = c_{1}\alpha \cdot \Phi\left(\Phi^{-1}\left(c_{1}\alpha\right) + \delta \right)\\ &\quad + c_{2}\alpha \left(1 - \Phi\left(\Phi^{-1}\left(c_{1}\alpha\right) + \delta \right)\right) \\ &\quad + \left(1 - c_{1}\alpha\right) \Phi\left(\Phi^{-1}\left(c_{2}\alpha\right) + \delta \right), \end{array} $$

The optimal choice of critical constants is followed by solving

15 $$\begin{array}{*{20}l} \text{maximize}_{c_{1}, c_{2}} \quad &\text{Power}\left(c_{1},c_{2}\right) \\ \text{subject to} \quad &\left(1 - 2c_{2}\right) + c_{1}\left(2c_{2} - c_{1}\right)\alpha = 0.  \end{array} $$

This optimization problem has no explicit solution. When *δ*=2.88 and *α*=0.05, the optimal solution is (*c*_1_,*c*_2_)=(1.0076,0.4998). While the step-function based *p*-value model and the normal-distribution based *p*-value model produce similar choices of critical constants (*c*_1_,*c*_2_), the model based on step function has an explicit solution of critical constants and requires little computational effort.

## Discussion and conclusions

We have given a step-function-based *p*-value model and its simplified version. These *p*-value models are simple and concise to perform theoretical power analysis. In addition, different test statistics, for example, *t*, chi-square or *F*, can be transformed to the *p*-value scale. These models can be applied to the field of multiple testing procedures to explain the assumption of monotonicity of the critical constants. We also use these *p*-value models to choose suitable sets of critical constants with more power. In this paper, we consider the independent *p*-values for the multivariate cases. Dependence structures can be brought into these *p*-value models, like Sarkar et al. [23] or Gou and Tamhane [13], and we will report the dependent multivariate *p*-value models in a separate paper. Finally, there are many applications of the step-function-based *p*-value models, and multiple testing procedure is an example in point.

## Abbreviations

BMI: Body mass index
SE-eating: Self-efficacy for controlled eating

## References

[1] Alosh M, Huque MF (2010). A consistency-adjusted alpha-adaptive strategy for sequential testing. Stat Med.

[2] Annesi JJ, Johnson PH, Tennant GA, Porter KJ, McEwen KL (2016). Weight loss and the prevention of weight regain: evaluation of a treatment model of exercise self-regulation generalizing to controlled eating. Permanente J.

[3] Bauer P (1989). Sequential tests of hypotheses in consecutive trials. Biom J.

[4] Bretz F, Hothorn T, Westfall P. Multiple Comparisons Using R; 2010.

[5] Bretz F, Maurer W, Brannath W, Posch M (2009). A graphical approach to sequentially rejective multiple test procedures. Stat Med.

[6] Burman CF, Sonesson C, Guilbaud O (2009). A recycling approach for the construction of Bonferroni-based multiple tests. Stat Med.

[7] Cai G, Sarkar SK (2008). Modified Simes’ critical values under independence. Stat Probab Lett.

[8] Feise RJ (2002). Do multiple outcome measures require *p*-value adjustment. BMC Med Res Methodol.

[9] Finner H, Roters M (1994). On the limit behavior of the joint distribution of order statistics. Ann Inst Stat Math.

[10] Finner H, Gontscharuk V (2009). Controlling the familywise error rate with plug-in estimator for the proportion of true null hypotheses. J R Stat Soc Ser B.

[11] Gou J, Tamhane AC (2014). On generalized Simes critical constants. Biom J.

[12] Gou J, Tamhane AC, Xi D, Rom D (2014). A class of improved hybrid Hochberg-Hommel type step-up multiple test procedures. Biometrika.

[13] Gou J, Tamhane AC. Hochberg procedure under negative dependence. 2015. Technical report, Department of Statistics, Northwestern University, Evanston, Illinois.

[14] Hayter AJ, Tamhane AC (1991). Sample size determination for step-down multiple test procedures: orthogonal contrasts and comparisons with a control. J Stat Plan Infer.

[15] Hochberg Y, Tamhane AC (1987). Multiple Comparison Procedures.

[16] Hsu JC (1988). Sample size computation for designing multiple comparison experiments. J Comput Stat Data Anal.

[17] Hung HM, O’Neill RT, Bauer P, Köhne K (1997). The behavior of the *p*-Value when the alternative hypothesis is true. Biometrics.

[18] Jennison C, Turnbull BW (2000). Group Sequential Methods with Applications to Clinical Trials.

[19] Jung S, Bang H, Young S (2005). Sample size calculation for multiple testing in microarray data analysis. Biostatistics.

[20] Lazzeroni LC, Ray A (2012). The cost of large numbers of hypothesis tests on power, effect size and sample size. Mol Psychiatry.

[21] Maxwell SE, Kelley K, Rausch RJ (2008). Sample size planning for statistical power and accuracy in parameter estimation. Annu Rev Psychol.

[22] Rom DM (1990). A sequentially rejective test procedure based on a modified Bonferroni inequality. Biometrika.

[23] Sarkar SK, Fu Y, Guo W (2016). Improving Holm’s procedure using pairwise dependencies. Biometrika.

[24] Sarkar SK, Guo W, Finner H (2012). On adaptive procedures controlling the familywise error rate. J Stat Plan Infer.

[25] Simes RJ (1986). An improved Bonferroni procedure for multiple tests of significance. Biometrika.

[26] Šidák Z (1967). Rectangular confidence regions for the means of multivariate normal distributions. J Am Stat Assoc.

[27] Stange J, Bodnar T, Dickhaus T (2015). Uncertainty quantification for the family-wise error rate in multivariate copula models. AStA Adv Stat Anal.

